# Pregnancy loss in cattle with emphasis on embryo transfer programs

**DOI:** 10.1590/1984-3143-AR2025-0045

**Published:** 2025-08-05

**Authors:** Roberto Sartori, Mirela Balistrieri, Lucas Oliveira e Silva, Carlos Eduardo Cardoso Consentini, Leonardo de França e Melo, Guilherme Correa de Sousa Pontes, Douglas Gaitkoski

**Affiliations:** 1 Departmento de Zootecnia, Escola Superior de Agricultura Luiz de Queiroz, Universidade de São Paulo – USP, Piracicaba, SP, Brasil; 2 GlobalGen vet science, Jaboticabal, SP, Brasil; 3 Departamento de Zootecnia, Universidade Federal de Goiás – UFG, Goiânia, GO, Brasil; 4 Rehagro, Belo Horizonte, MG, Brasil; 5 CPEx Embriões, Mogi Mirim, SP, Brasil

**Keywords:** embryo transfer, embryonic death, fertility, reproductive failures

## Abstract

Pregnancy loss (PL) in cattle significantly impacts reproductive efficiency and economic viability of herds. Of particular interest, PL in *in vitro* embryo production (IVP) systems, represents a major challenge to the success of this technique. Establishment and maintenance of pregnancy is influenced by factors such as fertilization, maternal environment, and embryonic signaling issues. Data on dairy cattle have shown that embryo transfer (ET) may lead to greater initial pregnancy, but greater PL compared to artificial insemination (AI), and the impact of environmental conditions on reproductive outcomes seems to be manageable with proper heat stress mitigation strategies, for example. Data on beef cattle submitted to IVP and ET have shown that recipient cows had greater pregnancy per ET (P/ET) and lower PL compared to recipient heifers, with sex-sorted sperm yielding similar or even greater P/ET than conventional semen. Distinct synchronization protocols for recipients yield different reproductive outcomes, and recipient breed also affects P/ET and PL. Moreover, embryo recipients that express estrus after synchronization, as well as recipients in which better quality embryos are transferred, tend to have greater P/ET and lower PL. These findings highlight the importance of management strategies to improve fertility and reduce PL in embryo recipients.

## Introduction

The success of reproductive programs, both in dairy and beef herds, is highly dependent on reproductive efficiency, which directly influences herd productivity and economic viability. In this context, pregnancy loss (PL) represents a major challenge, impairing reproductive outcomes and reducing profitability. The economic impact of PL has been studied. In dairy herds, De Vries (2006) estimated that each confirmed pregnancy contributes with ~US$278 to overall farm income, whereas each PL results in an estimated deficit of US$555 – values that vary depending on the stage of gestation and timing within lactation. In beef herds, although individual PL are less frequently quantified, reproductive failures still incur substantial economic burdens. Mercadante et al. (2020) projected a loss of US$6.25 per exposed female for each 1% decrease in pregnancy rates in artificial insemination (AI) programs. When extrapolated to large-scale systems, these incremental losses can accumulate to over US$2.8 billion annually in the U.S. beef industry (Mercadante et al., 2020). It is important to note that these values are not directly comparable due to differences in calculation approaches (individual PL in dairy vs. aggregated pregnancy rate reductions in beef), yet both highlight the major economic consequences of PL across production systems.

The PL in bovine females can be classified based on the stage of pregnancy in which it occurs. During the embryonic period, from fertilization to the end of embryonic cell differentiation (~42 days), PL is classified as early (before “maternal recognition of pregnancy”) or late (after “maternal recognition”) embryonic loss. Once the fetal stage begins, losses are classified as fetal mortality (Gábor et al., 2016). Despite the high fertilization rates − around 90% in naturally bred or artificially inseminated beef females and dairy heifers, and approximately 78% in high-producing dairy cows (Santos et al., 2004; Diskin et al., 2016) − there is a progressive reduction in the number of females maintaining pregnancy until calving, leading to low calving rates, sometimes below 30%. Munhoz et al. (2025) reported 12.8% PL between days 31 and 62 of pregnancy, and 12.1% from day 120 to calving, in cows that were inseminated after timed AI (TAI). These incidences were greater than PL from day 62 to 120 (6.4%). Interestingly, lower serum pregnancy-associated glycoprotein (PAG) concentrations on day 31 were associated with greater PL, suggesting that PAG could be a valuable biomarker for early identification of reproductive failures. These findings emphasize the need for early and accurate pregnancy detection to mitigate reproductive losses and improve herd productivity.

The greatest incidence of PL occurs during the early embryonic period, within the first few weeks of pregnancy (Wiltbank et al., 2016; Reese et al., 2020). Domingues et al. (2023) reported that between days 20 and 33, PL in dairy cows was evenly split between two causes: corpus luteum (CL) regression and conceptus failure, the latter indicated by a decline in PAG concentrations, despite stable progesterone (P4) concentrations. Given that pregnancy diagnosis is typically performed around 30 days after AI, most early losses are not detected, making it difficult to quantify their incidence, to identify underlying causes, and to implement mitigation strategies. Otherwise, although more easily detected, late embryonic or fetal loss remain largely overlooked despite their significant impact on reproductive efficiency and economic returns.

In the context of *in vitro* embryo production (IVP), PL is equally or even more critical, as the process introduces additional biological and technical challenges that can influence embryo survival, representing a limiting factor for the success of this biotechnology. The ability to establish and maintain pregnancy can be affected by multiple factors, including gamete quality (oocytes and sperm), fertilization success, embryo viability, uterine receptivity, endocrine environment, and maternal capacity to maintain pregnancy until calving (Sartori et al., 2004, 2010; Atkins et al., 2013; Wiltbank et al., 2016). Given the substantial investments in IVP programs, understanding the mechanisms underlying PL and developing strategies to minimize its occurrence are essential to optimize reproductive efficiency and profitability of herds. This review aims to synthesize and discuss findings from various studies and databases on fertility and PL in cattle IVP systems, focusing on its incidence and potential causes.

## Early embryonic development

The early stages of embryonic development are critical for properly establishing and maintaining pregnancy. Following fertilization, the embryo moves from the oviduct to the uterus at the 8- to 16-cell stage (Grealy et al., 1996). By the 5^th^ to 6^th^ day of development, the embryo reaches the 16- to 32-cell stage, and these cells begin to cluster, forming a compact sphere known as a morula. This marks the first critical stage of pregnancy, in which the embryo starts to act as an individual organism. On the 7^th^ or 8^th^ day, a fluid-filled cavity (blastocoel) begins to form, and the blastocyst differentiates into two main cell types: the inner cell mass, which gives rise to the embryo, and the trophoblast, which contributes to the formation of the placenta. This cellular organization is well described by Sreenan et al. (2001). Between the 9^th^ and 10^th^ day, the expanded blastocyst hatches from the zona pellucida and continues to expand before beginning to elongate around the 13^th^ day. Elongation occurs near the time of “maternal recognition of pregnancy” and is characterized by increased metabolic activity and secretion of interferon-Tau (IFN-τ; Thatcher et al., 2001). In the bovine, attachment of the extraembryonic membranes to the endometrium begins around day 19 and is completed by day 42, marking the end of the embryonic period and the completion of the cellular differentiation. Embryo survival, as well as the establishment and maintenance of pregnancy, involves active and passive communication between the embryo and the uterus. Around the 15^th^ day, the presence and signaling of the embryo in the uterus activate luteoprotective mechanisms, temporarily controlling the synthesis and release of PGF2α by the endometrium (Geisert et al., 1994; Mann et al., 1999; Thatcher et al., 2001; Okuda et al., 2002), thereby preventing luteolysis and ensuring the maintenance of the CL and continued P4 production, which is required for preparing the endometrium for implantation and proper embryonic development.

## Stages of pregnancy loss

Embryonic mortality can result from inherent defects in the embryo, an inadequate maternal environment, asynchrony between the embryo and the dam, or dam's failure to respond appropriately to the embryo's signals (Hansen, 2002). Wiltbank et al. (2016) described four critical periods of PL in the first trimester of gestation. The first period (the first week after AI or mating) involves fertilization failures or interruption in early embryonic development. The second period (between days 8 and 27 of pregnancy) is characterized by losses caused by uterine environment alterations, failures in embryonic signaling processes, problems with maternal recognition of pregnancy, or failure in the mechanisms that maintain the CL. In the third period (days 28 to 60 of pregnancy), losses are mainly due to failures in placentation and embryonic/fetal underdevelopment. Finally, the fourth period (days 61 to 90 of pregnancy) is predominantly associated with diseases and twin pregnancies, especially when two fetuses develop in the same uterine horn. A fifth period exists, involving fetal death after day 90 of pregnancy, which is often linked to infectious diseases, and severe nutritional, environmental, or metabolic disorders.

Although several studies reported high fertilization rates after AI or mating in cattle (Santos et al., 2004; Diskin et al., 2016), the pregnancy outcomes observed during the first pregnancy confirmation (usually performed on day 28-32) indicates high incidence of embryonic mortality during the early stages of pregnancy. In line with previous studies (Wiltbank et al., 2016; Berg et al., 2022; Albaaj et al., 2023; Crowe et al., 2024, 2025), the majority of embryonic mortality occurred early in gestation. For instance, a meta-analysis by Reese et al. (2020) reported 47.9% of PL during the early embryonic period (defined in their study as the period between fertilization and day 28 of pregnancy), with 28.4% occurring in the first week, 3.9% between days 7 and 16 of pregnancy, and 15.6% between days 16 and 32.

Experimental models commonly used to evaluate early pregnancy and embryonic mortality in cattle are based on slaughtering females at specific periods after AI to collect embryos/oocytes from the oviduct or uterus, or through *in vivo* embryo collection via uterine flushes (Boyd et al., 1969; Ayalon, 1978; Diskin and Sreenan, 1980; Roche et al., 1981; Maurer and Chenault, 1983; Sartori et al., 2002). More recently, new studies have focused on identifying and validating potential biomarkers and methods for early pregnancy diagnosis. Some of these biomarkers are related to the measurement of crown-rump length via ultrasonography, circulating concentrations of PAG (Pohler et al., 2016; Pursley et al., 2023; Santos et al., 2023; Munhoz et al., 2025), changes in the expression of mRNA and IFN-τ-stimulated gene (ISG) transcripts, and circulating P4 concentrations.

## Factors associated with pregnancy losses in cattle after *in vitro* embryo production

### Embryo quality

The classification of embryo quality is an intrinsic and essential process during IVP and embryo transfer (ET). The analysis of two large datasets (Sala et al., unpublished) of Holstein heifers receiving IVP embryos, evaluating pregnancy diagnosis on days 33 and 60, provided consistent results related to embryo quality (Table 1). In the first dataset (n = 5,092 ET), embryos grade 1 and 2, according to the International Embryo Technology Society guidelines (Stringfellow and Givens, 2010), had similar pregnancy per ET (P/ET) on d 33 (48.1 vs. 45.6%). However, PL was 70% greater for grade 2 compared to grade 1 embryos (25.6 vs. 15.1%; P < 0.05). In the second dataset (n = 12,569 ET), a difference in P/ET on day 33 was detected, with greater fertility in heifers receiving grade 1 embryos (48.5 vs. 39.6%), while PL was still higher for grade 2 embryos (23.6 vs. 19.6%).

**Table 1:** Pregnancy per embryo transfer (P/ET) and pregnancy loss in Holstein heifers with grade 1 and 2 *in vitro*-produced embryos. Data from Sala et al. (unpublished).

**Item**	**Dataset 1**		**P-value**	**Dataset 2**		**P-value**
	**Grade 1**	**Grade 2**		**Grade 1**	**Grade 2**	
n	4,573	519	-	7,952	4,617	-
P/ET on day 33, %1	48.1	45.6	0.12	48.5	39.6	<0.0001
Pregnancy loss, %2	15.1	25.6	0.04	19.6	23.6	0.002

^1^Pregnancy diagnosis was performed 26 days after ET; ^2^Pregnancy loss between day 26 and 59 after ET.

### Health problems

The effect of diseases during early lactation on fertility and PL of lactating dairy cows receiving AI or TAI has been extensively reported. Both uterine and non-uterine diseases are associated with lower P/AI and higher risks for PL, not only in cows receiving the first postpartum service but also in those receiving AI up to 200 days in milk (DIM), consequently reducing reproductive performance and calving rate (Ribeiro et al., 2016; Carvalho et al., 2019). The study by Ribeiro et al. (2016), including dairy cows receiving the first AI postpartum and with a single ovulation after AI, reported that the occurrence of diseases before AI reduced the proportion of cleaved, live (grades 1 to 3) and high-quality (grade 1 and 2) embryos. In addition, on days 15-16 after AI, the length of the recovered conceptus was smaller and the concentration of IFN-τ in the uterine flush was lower in cows experiencing diseases compared to healthy cows (Ribeiro et al., 2016). The negative effects of health problems on fertility and PL (between the first and second pregnancy diagnosis, or up to calving) was also described in studies including lactating dairy cows submitted to transfer of fresh IVP embryos, frozen IVP embryos, or frozen *in vivo*-derived (IVD) embryos from superstimulated donor cows (Ribeiro et al., 2016; Edelhoff et al., 2020), indicating negative effects of diseases in uterine level, early embryo development, and pregnancy maintenance.

In the study by Edelhoff et al. (2020), several factors were identified as significant predictors of uterine and nonuterine diseases in dairy cows. For uterine diseases, such as retained placenta, metritis, and endometritis, multiparity and calving during the cool season were associated with increased risk. Similarly, the risk of nonuterine diseases, including mastitis, pneumonia, lameness, and displaced abomasum, was markedly higher in multiparous cows. The same study demonstrated that both uterine and nonuterine diseases negatively affect synchronization rate, P/ET on day 31, and PL from day 31 to 59 on the first ET. Moreover, to the second ET, the nonuterine disease had a significant effect on fertility. These findings highlight the critical impact of health on fertility outcomes and emphasize the need for preventive and early intervention strategies to improve reproductive efficiency, especially in cows undergoing ET.

### Expression of estrus

One important physiological parameter associated with synchronization rate and fertility in synchronization programs is the expression of estrus. Cows expressing estrus have greater ovulatory follicles, ovulation rates, and higher subsequent circulating P4 and PAG, all associated with increased fertility (Mann and Lamming, 1999; Forde et al., 2011; Rizos et al., 2012; O’Hara et al., 2014; Forde and Lonergan, 2017). Achieving high ovulation rate is crucial for fertility in TAI, and the number of recipients available for timed-ET (TET) programs, and expression of estrus is associated with greater ovulatory response (Consentini et al., 2025). Regarding TET, Cedeño et al. (2020), using different synchronization protocols reported greater proportion of nonlactating multiparous beef recipients available for ET if they had expressed estrus, besides greater P/ET and pregnancy maintenance after transfer of IVD or IVP embryos. In lactating dairy cows, Pereira et al. (2016) reported that expression of estrus at the end of the synchronization protocol for transfer of fresh IVP embryos, was associated with greater P/ET on day 32 (46.2 [645/1,397] vs. 32.7% [193/606]) and reduced PL (18.6 [120/645] vs. 32.7% [43/193]). These results indicate the importance of circulating estradiol and expression of estrus prior to ovulation in recipients, suggesting potential positive effects in uterine environment, embryo development, and pregnancy maintenance.

### Circulating progesterone during the pre-ovulatory follicular wave

The presence of CL and circulating P4 concentrations at the beginning of a synchronization protocol and at the time of PGF2α treatment (at the end of the protocol) is associated with greater fertility and reduced PL. The presence of CL and higher P4 concentrations during the time of follicle development in TAI protocols is related to improved oocyte and embryo quality, and lower multiple ovulation rates, both associated with greater fertility and reduced PL (Ahmad et al., 1994; Revah and Butler, 1996; Wiltbank et al., 2011; Martins et al., 2018; Monteiro et al., 2021). Regarding embryo production and quality, Rivera et al. (2011) reported that cows yielding over 40 kg/day of milk that had their ovaries superstimulated to produce multiple ovulations during the first follicular wave (lower P4 concentrations during follicle growth) had greater percentage of degenerate embryos (23.5%) when compared with cows with superstimulated ovaries during the first follicular wave but with P4 supplementation (7.1%), or those with superstimulated ovaries during the second follicular wave, under higher P4 concentrations (3.9%). Moreover, the percentage of transferable embryos was considerably greater after superstimulation during the second follicular wave (88.5%), and the first wave with supplementary P4 (78.6%) than superstimulation during the first follicular wave (55.9%). Interestingly, the circulating P4 during the TET protocol, specifically at the time of PGF2α treatment, has also been associated with pregnancy maintenance in lactating dairy cows. For instance, Pereira et al. (2016) reported greater P/ET on day 32 and 60 in lactating dairy cows receiving fresh IVP embryos when P4 at the time of PGF2α treatment was ≥ 3.66 ng/mL compared to < 3.66 ng/mL. Additionally, a tendency for a linear effect of circulating P4 concentrations at the time of treatment with PGF2α on probability of pregnancy on day 60 was observed. These results suggest that, although the effects on oocyte quality and multiple ovulations are expected to not affect fertility of embryo recipients, the P4 environment during de preovulatory follicular wave has potential effects on the fertility of embryo recipients.

### Circulating progesterone after ovulation and during early pregnancy

The requirement for P4 after ovulation for pregnancy maintenance is well known and was demonstrated over 100 years ago (Fraenkel and Cohn, 1901; Magnus, 1901). Recent studies have reported that higher circulating P4 concentrations at the time of ET were associated with higher ISG expression, pregnancy-specific protein B (PSPB) concentrations and more pregnancies from day 18 up to calving (Crowe et al., 2024). Although elevated concentrations of P4 in the period immediately following conception have been associated with advancement of conceptus elongation, increase in IFN-τ production, and greater conception rates in cattle, the effects of different strategies attempting to increase circulating P4 after ovulation on fertility has not been consistent among studies (Lonergan and Sánchez, 2020).

A study from our laboratory (Monteiro et al., 2015) treated recipient lactating dairy cows with an intravaginal P4 device (1.9 g) before ET to increase circulating P4 concentrations. The experiment included three groups: (1) a control group, in which cows were not treated with P4 (control; n = 132), (2) cows receiving a P4 device 4 days before ET, with the device removed at the time of ET (ET-CIDR-4; n = 119), and (3) cows receiving a device 4 days before ET, and kept until 10 days after ET (ET-CIDR-14; n = 109). In this study, P4-supplemented lactating dairy cows that received an IVP embryo had a substantial decrease in P/ET on days 32 and 88 compared with the control group. On day 32, P/ET was greater (P < 0.001) in the control group (39.7%) compared to ET-CIDR-4 (21.3%), or ET-CIDR-14 (15.2%) groups. A similar pattern (P < 0.001) was observed on day 88 (control: 27.9%, ET-CIDR-4: 11.5%, and ET-CIDR-14: 11.7%). However, pregnancy loss between Days 32 and 88 did not differ (P = 0.27) among treatments (control: 29.5%, ET-CIDR-4: 45.7%, and ET-CIDR-14: 22.6%).

A strategy to increase circulating P4 during early pregnancy is by generating an accessory CL after an ovulation induced by GnRH or hCG at different stages of early pregnancy. A meta-analysis including studies with cows receiving AI evaluated the effect of several variables on fertility and their interactions with GnRH or hCG treatment at different days (Besbaci et al., 2020). The results suggest certain benefits of this strategy, such as an increase in conception rate of cows with considered poor to moderate fertility (P/AI < 30 to 45%), but other factors should be considered such as parity, day and dose of treatments (Besbaci et al., 2020). Similarly, a meta-analysis including 12 studies with ET fertility results (differing in parity, average fertility, treatment type, dosage and time, etc.), reported benefits on fertility in specific cases, such as in cows with lower fertility (< 40%), however, no effect on PL was reported (Chen et al., 2023).

A recent study with ET recipient dairy heifers (n = 1,071), primiparous (n = 691), and multiparous cows (n = 379) evaluated the effect of GnRH or hCG treatment on the day of transfer of IVP embryos (El Azzi et al., 2023). The overall P/ET on day 37 was relatively high in that study for all categories (heifers: 67.7%, primiparous: 51.4%, and multiparous: 44.9%), as well as the PL up to calving (heifers: 14.6%, primiparous: 15.2%, and multiparous: 17.7%). However, no effect of GnRH or hCG treatment was detected on P/ET, calving/ET or PL. Another study (García-Guerra et al., 2020), with nulliparous heifers treated or not with GnRH on day 5 after the final GnRH of a 5-day synchronization protocol, reported greater circulating P4 concentrations on days 12 and 21 in treated heifers with accessory CL present, in addition to greater total CL volume on day 33. Nevertheless, no effect of treatment was detected on PSPB concentrations on day 28, or in overall P/ET and PL after transfer of IVP embryos. When evaluating the effect of treatment within embryo stage (blastocyst or expanded blastocyst), although unclear explanatory mechanisms, GnRH treatment and presence of accessory CL reduced PL between days 33 and 60 in heifers receiving expanded blastocysts. Regarding the side of the accessory CL (ipsi or contralateral), a study from our research group in lactating dairy cows receiving TAI demonstrated that cows treated with GnRH and having an ipsilateral accessory CL had ~50% lower PL than the control group of cows not receiving GnRH (6.6 vs. 13.7%). New studies are being currently performed with lactating Holstein cows receiving IVP embryos to evaluate the effect of the side of the accessory CL on circulating P4, PAG, fertility, and PL up to calving.

In accordance with literature reports, particularly regarding fertility and PL in both dairy and beef cattle, with an emphasis on ET, the following sections present similar results from two large-scale, retrospective databases. While the considerable sample size enhances the reliability of the observations, it is important to acknowledge that these findings are from observational datasets, not controlled experimental designs and should, therefore, be interpreted with appropriate caution.

## Fertility and pregnancy loss outcomes in dairy cattle

### Dataset characterization

A retrospective analysis was conducted by our laboratory using data from 10 commercial dairy herds implementing both TAI and ET programs between 2018 and 2023 (Vandresen et al., 2024). The dataset included reproductive records from heifers (AI: n = 18,681; ET: n = 5,341) and lactating dairy cows (AI: n = 42,036; ET: n = 9,120). All embryos transferred in the ET programs were IVP. To ensure data reliability, only sires and technicians with a minimum of 100 repetitions within the dataset were included in the analyses. Reproductive performance was evaluated based on P/AI or P/ET, determined between 28 and 40 days post-breeding, and PL between first pregnancy diagnosis and calving.

### Effects of biotechnology: TAI vs. ET

According to this dataset, heifers had greater pregnancy when submitted to ET, whereas PL was substantially greater in ET (Table 2). In cows, conception rate was similar between biotechnologies, but incidence of PL was greater for ET than AI (Table 2). As a result, in both heifers and cows, the calving rate was greater when females were submitted to AI compared to ET with IVP embryos (Table 2). These results coincide with previous studies indicating that, although ET can result in greater initial conception rate, it is also associated with a greater risk of PL due to factors such as embryo quality, uterine environment, and “maternal recognition of pregnancy” (Hansen, 2002; Pohler et al., 2016), which results in a lower number of calves born.

**Table 2:** Conception rate, pregnancy loss, and calving rate in dairy cattle undergoing artificial insemination (AI) or embryo transfer (ET) with *in vitro*-produced embryos. Data from Vandresen et al. (2024).

**Biotechnology within category**	**Conception rate 1 , % (n)**	**Pregnancy loss 2 , % (n)**	**Calving rate, % (n)**
**Heifers**			
AI	38.0 (18,681)	18.4 (7,093)	31.0 (18,681)
ET	41.4 (5,341)	35.7 (2,211)	26.6 (5,341)
P-value	0.01	< 0.01	< 0.01
**Cows**			
AI	36.4 (42,036)	34.2 (15,290)	23.9 (42,036)
ET	33.9 (9,120)	47.7 (3,094)	17.7 (9,120)
P-value	0.20	< 0.01	< 0.01

^1^Pregnancy diagnosis was performed between 28 and 40 days after AI or between 21 and 33 days after ET; ^2^Pregnancy loss between first pregnancy diagnosis and calving.

The season did not influence reproductive outcomes in this dataset, with conception rates (P/AI and P/ET) having no significant variation between warm and cool seasons, neither in heifers (P/AI: warm: 38.1% [3,536/9,277] and cool: 37.8% [3,557/9,404]; P/ET: warm: 41.2% [1,035/2,511] and cool: 41.6% [1,176/2,830]), nor cows (P/AI: warm: 35.8% [7,524/21,022] and cool: 37.0% [7,776/21,014]; P/ET: warm: 33.9% [1,489/4,388] and cool: 33.9% [1,605/4,732]). Pregnancy loss remained similar across seasons in heifers (AI: warm: 18.7% [661/3,536] and cool: 18.1% [644/3,557]; ET: warm: 35.0% [362/1,035] and cool: 36.4% [428/1,176]) and cows (AI: warm: 35.0% [2,633/7,524] and cool: 33.5% [2,605/7,776]; ET: warm: 47.4% [706/1,489] and cool: 48.0% [770/1,605]). These findings are according to other studies that suggest that although heat stress is a well-documented cause of reproductive failure, its impact on pregnancy maintenance may be mitigated by appropriate management strategies (Wiltbank et al., 2016).

Parity was an important factor affecting pregnancy success in both biotechnologies. Regardless of biotechnology, pregnancy decreased as parity increased (Table 3), with primiparous cows having the greatest (P ≤ 0.05) conception rate (36.9%^a^ [9,139/24,768]), followed by second lactation cows (35.3%^b^ [5,220/14,788]), and multiparous cows (34.6%^c^ [4,014/11,600]). Specifically, among cows receiving ET, P/ET was greater (P ≤ 0.05) in primiparous (Table 3), which did not differ from each other. In addition, regardless of biotechnology, PL was greater (P ≤ 0.05) in multiparous cows (37.6%^a^ [1,510/4,016]), and secundiparous (37.5%^a^ [1,959/5,224]) than in primiparous cows (35.4%^b^ [3,237/9,144]). These results are consistent with findings from Munhoz et al. (2025), who reported that multiparous experience greater PL than primiparous cows, likely due to increased metabolic demands and cumulative reproductive stress (Chebel et al., 2004; Diskin et al., 2016). However, in this dataset, we did not observe an interaction effect between biotechnology and parity.

**Table 3:** Effects of parity on fertility in dairy cattle undergoing artificial insemination (AI) or embryo transfer (ET) with *in vitro*-produced embryos. Data from Vandresen et al. (2024).

**Parity**	**Biotechnology**		**P-value**		
	**AI**	**ET**	**Biotec. 3**	**Parity 4**	B*P 5
**Conception rate**1					
Primiparous, % (n)	37.0 (20,251)	36.6a (4,517)	0.20	< 0.01	< 0.01
Secundiparous, % (n)	36.3 (12,100)	30.9b (2,688)			
Multiparous, % (n)	35.2 (9,685)	31.9^b^ (1,915)			
**Pregnancy loss**2					
Primiparous, % (n)	33.1 (7,492)	46.0 (1,652)	< 0.01	< 0.01	0.89
Secundiparous, % (n)	35.3 (4,393)	49.1 (831)			
Multiparous, % (n)	35.2 (3,405)	50.4 (611)			

^a,b^Superscript letters denote differences (P ≤ 0.05) between rows within columns. ^1^Pregnancy diagnosis was performed between 28 and 40 days after AI or between 21 and 33 days after ET; ^2^Pregnancy loss between first pregnancy diagnosis and calving; ^3^Refers to the main effect of biotechnology; ^4^Refers to the main effect of parity; ^5^Refers to the interaction between biotechnology and parity.

The findings highlight the complexity of reproductive success in dairy cattle, with ET leading to greater initial conception rates in heifers and greater PL in cows and heifers compared to AI. Additionally, parity remains a crucial factor, with primiparous having greater fertility outcomes.

## Fertility and pregnancy loss in beef cattle

### Dataset characterization

The database was collected from August 2021 to February 2023 from a single IVP embryo company and donors from a single farm, and recipients from eight farms (unpublished data). To ensure data reliability, only sires with a minimum of 100 repetitions were included in the analysis. We evaluated the effects of donor category (Nelore donors: calf, heifer, or cow), type of semen (conventional or sex-sorted), protocol length (7, 8, or 9 days of intravaginal P4 device), breed (Nelore or crossbred [Angus × Nelore]), and recipient category (heifer, primiparous, or multiparous). For each response variable analyzed, a specific sample group was defined.

### Effect of donor category

To evaluate the effect of donor category, data from primiparous and multiparous recipients that received embryos from donors classified as calves, heifers, or cows were analyzed. This analysis considered the donor category, recipient category, semen type (conventional or sex-sorted), and their interactions.

The donor category influenced P/ET but not PL (Table 4). In Nelore recipients, heifer donors had greater (P ≤ 0.05) P/ET than calf donors, and cow donors did not differ from the others. In crossbred (Angus × Nelore) recipients, cow donors had greater (P ≤ 0.05) P/ET than calves or heifer donors. The donor category did not affect PL (Table 4) in Nelore or crossbred (Angus × Nelore) recipients (Table 4).

**Table 4:** Effects of donor category on pregnancy per embryo transfer (P/ET) and pregnancy loss in beef cattle undergoing *in vitro* embryo production. Unpublished data.

Recipient breed	**Donor category**			P-value
	**Calf**	**Heifer**	**Cow**	
P/ET, % (n)				
Nelore	38.5y	45.9^x^	44.2xy	0.019
	(1,073)	(1,029)	(925)	
Crossbred (Angus x Nelore)	39.9y	41.9^y^	48.4x	0.005
	(1,030)	(831)	(3,255)	
Pregnancy loss^1^, % (n)				
Nelore	12.1	15.0	8.6	0.17
	(413)	(472)	(409)	
Crossbred (Angus x Nelore)	24.3	15.5	19.0	0.25

^x,y^Superscript letters denote differences (P ≤ 0.05) within rows. ^1^Pregnancy loss between 30 and 90 days of pregnancy.

### Effect of type of semen

To evaluate the effects of semen type (Table 5), data from donor cows and primiparous and multiparous recipients were analyzed. This analysis considered the recipient category, recipient breed, semen type (conventional or sex-sorted), and their interactions.

**Table 5:** Effects of semen, synchronization protocol, and recipient on fertility (pregnancy per embryo transfer [P/ET]) in beef cattle undergoing *in vitro* embryo production (IVP). Unpublished data.

**Item**	**n**	**P/ET, %**	**Pregnancy loss 5 , %**
Type of semen1			
Conventional	2,063	46.0	15.9
Sex-sorted	2,117	48.9	17.7
P-value	-	0.02	0.83
Protocol length2			
7 days	335	64.8ª	10.6
8 days	950	52.6b	12.0
9 days	401	50.4^b^	17.8
P-value	-	0.0007	0.15
Recipient category3			
Heifer	1,339	39.1^b^	31.4ª
Primiparous cows	1,686	54.5ª	13.0^b^
Multiparous cows	230	58.3ª	11.9^b^
P-value	-	<0.0001	<0.0001
Recipient breed4			
Nelore	925	44.2	8.6
Crossbred (Angus x Nelore)	3,255	48.4	19.0
P-value	-	0.01	<0.0001

^a,b^Superscript letters denote differences (P ≤ 0.05) between rows within columns. ^1^The analysis of the type of semen was performed on a homogeneous sample universe, including only cow donors and cow recipients; ^2^The analysis of the protocol length was performed on a homogeneous sample universe, including only cow donors and crossbred (Angus × Nelore) primiparous recipients; ^3^The analysis of the recipient category was performed on a homogeneous sample universe, including only cow donors and crossbred (Angus × Nelore) recipients; ^4^The analysis of the breed recipients was performed on a homogeneous sample universe, including only donor cows and recipient cows; ^5^Pregnancy loss between 30 and 90 days of pregnancy.

The type of semen used for fertilization had a significant impact on fertility, with sexed semen achieving greater (P ≤ 0.05) P/ET compared to conventional semen (Table 5). However, PL did not differ between types of semen. Conversely, Palma et al. (2008) reported lower efficiency with sexed semen in IVP, showing reduced cleavage rates and blastocyst development. Similarly, Magata et al. (2021) observed dysmorphisms during the first cleavage and delayed embryo growth in bovine embryos produced with X-sorted sperm, which could impair the viability of embryos that reach the blastocyst stage. However, in our data, sexed semen enhanced initial conception rates without affecting pregnancy maintenance, possibly due to successful fertilization despite early developmental challenges. Additionally, evaluating early embryonic development and de-selecting embryos with abnormal first cleavage patterns could improve the success of IVP programs using sex-sorted sperm by selecting embryos with higher implantation potential.

### Effect of synchronization protocol length

**Figure 1:**
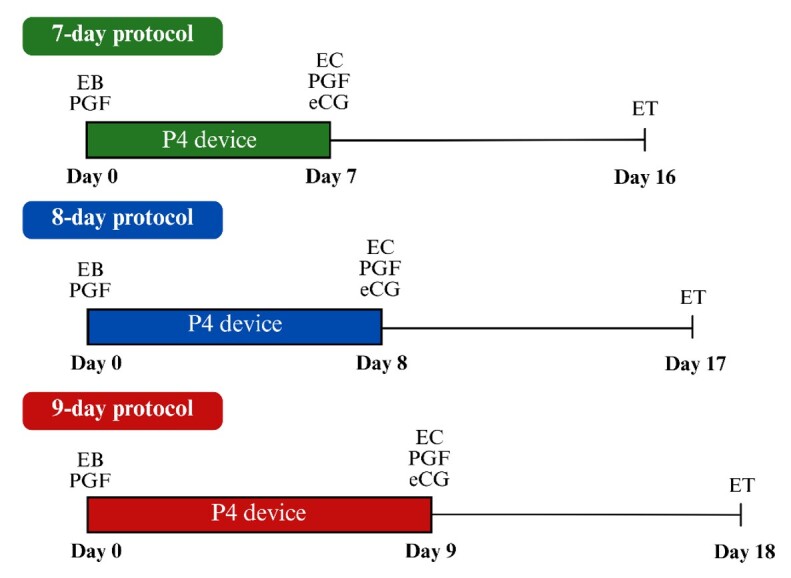
Schematic representation of synchronization protocols for embryo transfer (ET) with different lengths (7, 8, or 9 days) according to the duration of permanence of the intravaginal progesterone (P4) device. *EB: estradiol benzoate; PGF: prostaglandin-F2α analogue; EC: estradiol cypionate; eCG: equine chorionic gonadotropin.

To evaluate the effect of protocol length, data from donor cows and primiparous crossbred (Angus × Nelore) recipients were analyzed. The analysis considered the effect of intravaginal P4 device durations of 7, 8, or 9 days (Figure 1), type of semen (conventional or sex-sorted), and their interaction.

In these data, the length of hormonal synchronization protocols influenced P/ET outcomes (Table 5), with a 7-day protocol yielding the greatest (P ≤ 0.05) fertility compared with the longer protocols. Pregnancy loss did not differ (P = 0.15) among protocols with different times of permanence of the intravaginal P4 device (Table 5). A possible explanation for the superior performance of the 7-day protocol is that this duration better synchronizes the timing of ovulation, leading to a more synchronous uterus with embryo transferred at the time of ET. Additionally, according to personal communication, pregnancy success is greater when the embryo's developmental stage is more closely aligned with the recipient’s estrous cycle day. Therefore, the 7-day protocol may provide an advantage by improving this synchrony. Moreover, it is important to highlight that studies are still needed to confirm these associations and guide reproductive strategies based on solid evidence.

### Effect of recipient

To evaluate the effect of the recipient category, data from crossbred (Angus × Nelore) recipients receiving embryos from cows as donors were analyzed. The analysis considered recipient category (heifers, primiparous, or multiparous), semen type (conventional or sex-sorted), and their interaction. Additionally, to assess the effect of the recipient breed, only cows (primiparous and multiparous) were considered as recipients receiving embryos from cows as donors. This analysis included recipient breed (Nelore or Angus × Nelore), semen type, recipient category, and the interaction between recipient breed and semen type.

The recipient category affected P/ET and PL (Table 5). Multiparous and primiparous cows had greater (P ≤ 0.05) P/ET than heifers. Moreover, heifers had greater (P ≤ 0.05) PL than primiparous and multiparous cows (Table 5). These results emphasize the importance of selecting reproductively mature recipients to enhance ET success.

The recipient breed influenced pregnancy outcomes, with crossbred (Angus × Nelore) cows having the greatest (P ≤ 0.05) P/ET at 30 days of pregnancy compared to Nelore cows (Table 5). However, P/ET at 90 days of pregnancy was similar between breeds due to greater (P ≤ 0.05) PL in crossbred recipients compared to Nelore cows. These data show that although crossbred cows may have greater initial conception rates, Nelore cows maintain pregnancies more effectively, with no differences in final P/ET.

These data demonstrate that multiple factors influence the success of ET in beef cattle, with donor, semen, protocol, and recipient characteristics being pivotal. Cow donors and recipients significantly enhance pregnancy outcomes. Besides that, shorter synchronization protocols were associated with improved fertility rates, while despite greater initial P/ET in crossbred (Angus × Nelore) recipients, Nelore cows maintain pregnancies more effectively.

## Conclusion

The literature and database analyses converge to several key factors affecting reproductive success in cattle receiving IVP embryos. Embryo quality is a consistent determinant of pregnancy loss, with lower-quality embryos associated with reduced outcomes. Uterine and non-uterine health problems, lack of estrus, and abnormal hormonal environments, particularly lower circulating progesterone and estradiol, are associated with impaired embryo development and pregnancy maintenance. In dairy cattle, parity and environmental conditions impact fertility, while in beef cattle, donor and recipient characteristics, synchronization protocols, and genetic are decisive for successful outcomes. Although the databases comprise large and representative populations, providing valuable insights into field conditions, the observational nature of these data requires cautious interpretation. Controlled studies remain essential to validate these associations and inform evidence-based reproductive strategies.

## Data Availability

Research data is only available upon request.
